# Isolated pancreatic tuberculosis masquerading as pancreatic cancer

**DOI:** 10.1093/gastro/gou017

**Published:** 2014-03-19

**Authors:** George S. Zacharia, Rajany Antony, Sandesh Kolassery, Thazhath M. Ramachandran

**Affiliations:** Medical Gastroenterology, Government Medical College Calicut, India

**Keywords:** pancreatic tuberculosis, pancreatic cancer, endoscopic ultrasonography

## Abstract

Isolated pancreatic tuberculosis (TB) remains a rarity despite the high incidence of tuberculosis in many of the African and Asian countries. Presentation as discrete pancreatic mass often masquerades as pancreatic neoplasm and diagnosis may require histology. Extra-hepatic portal hypertension due to splenic vein thrombosis complicating pancreatic TB has been reported in the literature. We report here a case of isolated pancreatic TB with pancreatic head mass mimicking neoplasm with extra-hepatic portal hypertension. The possibility of TB should be considered in the list of differential diagnoses of pancreatic mass and an endoscopic, ultrasound-guided biopsy might help to clinch the diagnosis of this potentially curable disease.

## INTRODUCTION

Isolated pancreatic TB is an exceedingly rare condition, even in the developing world where the disease is endemic. However late cases are increasingly reported, probably due to further advancements in diagnostics. Presentation as discrete pancreatic mass often confuses the clinician and has a high likelihood of being falsely labelled as pancreatic neoplasm. We report here a case of isolated pancreatic TB presenting as pancreatic head mass with encasement and compression of the portal vein, causing left-sided portal hypertension with development of isolated gastric varices.

## CASE PRESENTATION

**Figure 1.**
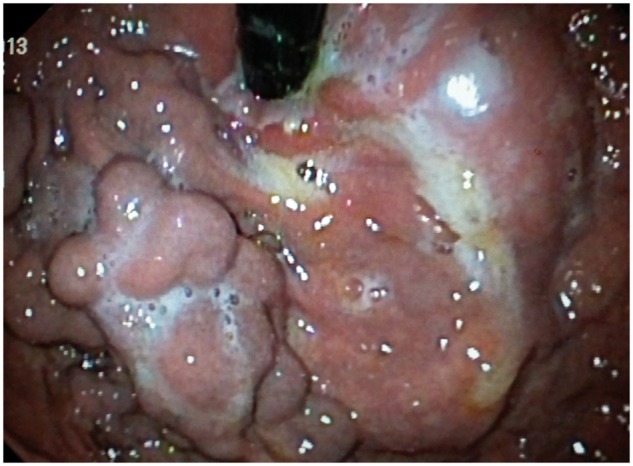
Gastroscopy in retroflexion, showing isolated gastric fundal varix: IGV1.

**Figure 2.**
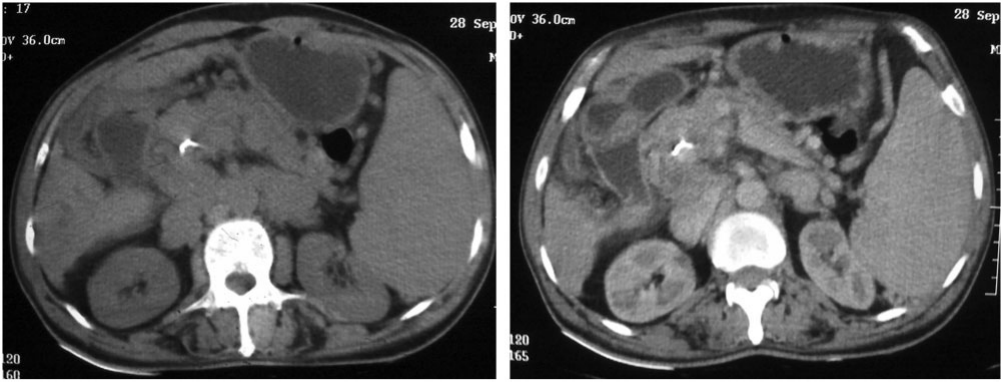
Plain and contrast CT images showing heterogeneously enhancing lesion in the pancreatic head region and calcification of portal vein.

**Figure 3.**
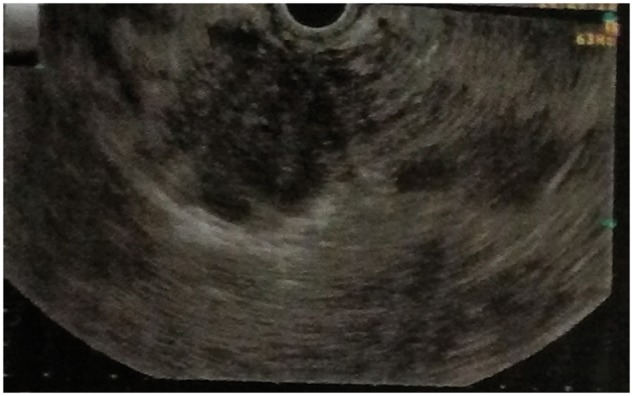
EUS image showing hypoechoic lobulated mass in relation to the pancreatic head, with anechoic areas and floating echogenic material within.

**Figure 4.**
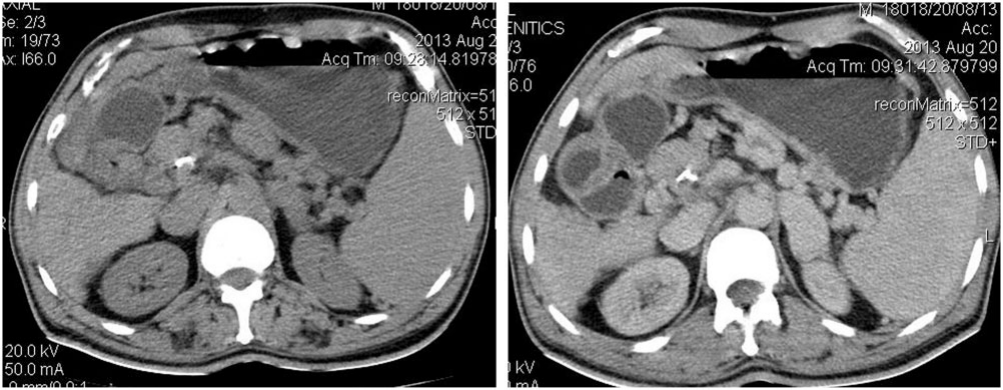
Follow-up CT images showing resolution of the mass. Portal vein calcification is seen persisting in the follow-up image.

A 60-year-old male patient presented with epigastric dull aching pain, anorexia and loss of weight of nine months duration. He had no history of vomiting, altered bowel habits, overt gastrointestinal bleed, fever, jaundice, no significant co-morbid illnesses nor any addictions. Examination was unremarkable, except for emaciation and a palpable spleen. Haemogram revealed anaemia (haemoglobin 9.6 g/dL), elevated erythrocyte sedimentation rate (100 mm/h) and thrombocytopenia (8.9 × 10^4^ cells/mm^3^). Serum electrolytes, renal and liver function tests were within normal limits. Oesophago-gastroduodenal endoscopy revealed isolated gastric fundic varix: Sarin’s classification IGV1 (Figure 1). Abdominal ultrasound revealed an enlarged spleen with dilated splenic vein (18 mm), multiple splenic hilar collaterals and a heteroechoic mass lesion in the pancreatic head. The liver and biliary tree were normal on sonogram. Contrast enhanced computerized tomography (CECT) of the abdomen showed a heterogeneously enhancing soft tissue density lesion in the pancreatic head extending to the porta and encasing the portal vein. Portal vein walls showed calcification within the lesion (Figure 2). Splenomegaly, multiple splenic and peripancreatic collaterals and a tiny peripancreatic lymph node were also noted. Serum CA 19-9 and IgG4 (for auto-immune pancreatitis) were within normal limits. Endoscopic ultrasound (EUS) revealed a hypoechoic lobulated mass in relation to the pancreatic head, with anechoic areas and floating echogenic material within it, suggestive of necrosis (Figure 3). The portal vein was narrowed, at 1.5 cm, in proximity to the mass lesion with calcification of the wall. Doppler ultrasound revealed the presence of venous blood flow through the narrowed portal vein segment, with multiple peripancreatic and perigastric venous collaterals. EUS-guided biopsy from the lesion revealed multiple granulomas, composed of epitheloid cells and occasional multinucleated giant cells. Sheets and clusters of lymphocytes, as well as background caseation necrosis, were also noticed. Aspirate smears were negative for acid-fast bacilli. The patient tested negative for human immunodeficiency virus (HIV) infection. A Mantoux test was strongly positive, while chest X-ray and AFB stain on induced sputum were non-contributory. The patient was started on antitubercular therapy (ATT), from which the patient had significant symptomatic improvement. Repeat CECT after completion of ATT showed resolution of the pancreatic head mass and the peripancreatic node; however, the collaterals were persistent (Figure 4).

## DISCUSSION

TB caused by mycobacterium tuberculosis is an endemic infectious disease in the developing world, with an estimated 9.7 million cases reported annually [1]. Although the lung is most commonly affected, in immunocompetent hosts extrapulmonary TB accounts for one-fifth of cases [2]. Pancreatic involvement is reported in less than 5% of cases and it often occurs in the setting of disseminated TB and immunodeficiency states [2, 3]. Isolated pancreatic TB is still rare, with only a handful of cases reported to date [4–6]. The disease often emerges insidiously with non-specific constitutional symptoms, while the most common presentations of pancreatic TB include abdominal pain, jaundice and weight loss [5, 7]. Patients may rarely present with gastro-intestinal bleed, secondary to splenic vein thrombosis [7]. Tuberculin skin testing may be beneficial, as it is reported to have sensitivity in the range of 58–100% in patients with abdominal TB [2]. Abdominal imaging may reveal solid or cystic lesions, typically in the pancreatic head; however the appearance is non-specific, since pancreatic neoplasms and pseudocysts can have similar appearances [2, 5, 7]. Clinical and radiological features being non-specific, histology is required for diagnosis. Available options for biopsy include percutaneous ultrasonography or computed tomography (CT)-guided biopsy, open or laparoscopic surgical biopsy and the EUS biopsy. Currently EUS biopsy is considered the ‘gold standard’ for diagnostic modality for pancreatic mass [5, 6]. Biopsy cytology in pancreatic TB shows granulomatous inflammation, epitheloid histiocytes, plasma cells and lymphocytes, while acid-fast bacilli are rarely seen [5, 8]. A positive mycobacterium tuberculosis culture is highly specific but is less sensitive and requires long incubation periods [8]. Once diagnosed, pancreatic TB is treated with standard ATT of at least six months' duration. Symptomatic response and repeat abdominal imaging guides the clinician regarding treatment response and duration [5].

Our patient presented with non-specific epigastric pain and constitutional features consistent with the published literature to date on pancreatic TB [4, 5, 7, 9]. He had non-specific markers of chronic inflammatory disease/TB in the form of anaemia, elevated ESR and a positive tuberculin skin test. There were no features of TB elsewhere and he was negative for HIV infection. Abdominal imaging had revealed a lesion in the pancreatic head with cytology consistent with TB. AFB staining was negative; however according to Farar *et al.*, nearly 40% of patients with abdominal TB can have negative AFB staining [10]. The relatively low yield of EUS biopsy might have caused the negative staining. Development of portal hypertension—as evidenced by splenomegaly, venous collaterals and isolated gastric varix—is exceedingly rare, although Saluja *et al.* had described splenic vein thrombosis complicating pancreatic TB [7]. Normal liver functions and imaging virtually rule out the possibility of an underlying chronic liver disease causing portal hypertension. The possibility of a patient with pre-existing portal vein thrombosis with partial recanalization developing superadded pancreatic TB cannot be ruled out; however the patient had no past history of gastro-intestinal bleeding or acute abdominal pain pointing towards such an event. Visualization of the portal vein and demonstration of portal venous flow through the narrowed portal vein segment is unlikely in portal vein thrombosis although the possibility of a partially recanalized thrombus in portal vein cannot be discounted with certainty, especially in the presence of portal vein wall calcification, which in turn can be a calcified remnant of the thrombus. The splenomegaly might have caused thrombocytopenia and contributed to anaemia. Pancreatic head neoplasm is a close differential diagnosis but the excellent response to ATT, tumour marker negativity and lack of disease progression over the last year of follow-up argues against this.

## CONCLUSION

Isolated pancreatic TB is an exceedingly rare clinical entity but should always be in the list of differential diagnoses in a patient with pancreatic mass, especially in the developing world, where the infection is endemic. Utilization of EUS with biopsy might aid in differentiating this benign disorder from pancreatic neoplasm.

**Conflict of interest:** none declared.
